# Distribution, Typical Structure and Self-Assembly Properties of Collagen from Fish Skin and Bone

**DOI:** 10.3390/molecules28186529

**Published:** 2023-09-08

**Authors:** Xuening Zhang, Jie Wang, Qian Zhang, Yan Fan, Hongwei Zhang, Khurshid Ahmad, Hu Hou

**Affiliations:** 1 College of Food Science and Engineering, Ocean University of China, Sansha Road, Qingdao 266404, China; zhangxuening23@163.com (X.Z.); wuaxueya@163.com (J.W.); a1104952517@163.com (Q.Z.); khurshidahmad694@yahoo.com (K.A.); 2 College of Marine Life Sciences, Ocean University of China, Yushan Road, Qingdao 266003, China; 3 Technology Center of Qingdao Customs, Xinyue Road, Qingdao 266109, China; light04@126.com; 4 Laboratory for Marine Drugs and Bioproducts, Laoshan Laboratory, Qingdao 266237, China; 5 Sanya Oceanographic Institution, Ocean University of China, Sanya 572024, China; 6 Qingdao Institute of Marine Bioresources for Nutrition and Health Innovation, Qingdao 266000, China

**Keywords:** collagen, self-assembly, unique peptide, structure

## Abstract

The source and type of collagen are crucial to its application, and both play a decisive role. Collagen was prepared from both tilapia skin and bone and skate skin and cartilage, named as CI-TI-s, CI-TI-b, CI-SK-s, and CII-SK-c, respectively. Types, distributions, structures, and self-assembly of collagen were studied. It showed that yellow collagen fibers from skin arranged longitudinally, while collagen fibers from skate cartilages displayed varying colors. CI-TI-s, CI-TI-b, CI-SK-s, and CII-SK-c showed the typical amide A (3316–3336 cm^−1^) and amide B (2929–2948 cm^−1^) in FTIR spectra. CI-TI-b and CII-SK-c showed 218–229 nm of UV absorption, 11.56–12.20 Å of d values in XRD, and 0.12–0.14 of Rpn values in CD. The thermal denaturation temperatures of CI-TI-s and CI-SK-s were 30.7 and 20.6 °C, respectively. The self-assembly of CI-TI-s and CII-SK-c were maximum at pH 7.2 and 7.4–7.6, respectively. The unique collagen peptides of tilapia and skate were GPSGPQGAVGATGPK, PAMPVPGPMGPMGPR, SPAMPVPGPMGPMGPR, GESGPSGPAGPAGPAGVR, SSGPPVPGPIGPMGPR, GLTGPIGVPGPPGAQGEK, GLAGPQGPR, and GLSGDPGVQGIK, respectively. The unique peptides of type I and type II collagen were GPTGEIGATGLAGAR, GVLGLTGMR, LGLTGMR, GEPGAAGPAGPSGPMGPR, SSGPPVPGPIGPMGPR, and GLSGDPGVQGIK, respectively.

## 1. Introduction

Collagen is mainly present in various skins, cartilages, tendons, and bones, and accounts for 30% of the protein content in mammals [1,2]. Type I collagen is the most abundant protein type in bones, skins, scales, and tendons, and it is the principal structural ingredient of connective tissues; this represents about 90% of total human collagen [3]. Type II collagen is usually present in cartilage, which is formed by combining collagen fibers with some proteoglycans [4]. It was reported that in Nile tilapia (*Oreochromis niloticus*), one of the most popularly cultured fish in China, the yields of collagen from skin were above 40% (dry weight) [5]. Skate (*Raja porosa*), a typical marine cartilaginous fish, was widely used in order to extract collagen from its cartilage.

There are many diversities in the function and application for different types of collagen. Type I collagen is often made into sponges and for wound dressing membranes [6,7]. Modified type I collagen is extensively used in bioprinting as a bio-ink due to its high compatibility with human cells [8]. In the pharmaceutical field, collagen I is used for drug delivery as nanospheres and nanoparticles [9]. In the food industry, type I collagen has been widely used as a food additive, as edible film, in drinks, and as a carrier [10]. Type II collagen can promote the differentiation of cartilage cells and improve bone health, and it is especially useful in the treatment of rheumatoid arthritis [11]. 

The structure and properties of collagen derived from fish have been extensively studied. These studies are important for determining the eventual application of collagen. The collagen α chain shows a repeated Gly-X-Y sequence, in which X and Y are generally Pro and Hyp, respectively [12]. Structural differences of collagen from different sources could not be found by FTIR, XRD, and UV spectroscopy [1]. Zhu et al. [4] found the Rpn of collagen from skate and sturgeon cartilage was 0.19–0.25 in CD, and the collagen exhibited obvious self-assembly with a concentration above 0.3 mg/mL, an adjustment of pH 7.4–7.6, and an NaCl concentration of 120 mmol/L. Zhang et al. [13] found that the denaturation temperatures of collagen from sturgeon scale, skin, and cartilage were 29.6, 26.8, and 36.3 °C, respectively. Romijn et al. [14] estimated the χ33/χ31-ratio for type I collagen in tendon and type II collagen in cartilage was 1.33 and 1.36, respectively. This was determined by polarization-resolved second harmonic, which cannot be easily used to differentiate collagen types. Therefore, collagen of different types and sources should be determined using other methods. Different collagen peptides could be identified using LC-MS/MS [15]. Zhang et al. [16] found diverse mixtures of collagen type II and I contained 12 specific peptides using LC-MS, which indicated that MS was a possible strategy for collagen type identification. Hu et al. [17] quantified 81 proteins of skipjack, bigeye, and yellowfin tuna by the sequential window acquisition of all theoretical fragment ion mass spectra (SWATH-MS) analysis and screened 14 protein biomarkers with the ability to distinguish the three tuna species.

In this work, collagens from skate and tilapia were extracted by acetic acid and pepsin, and then their structures and properties were assessed by UV, FTIR, CD, XRD, fractional viscosity, and self-assembly. The discovery of unique peptides of sources (tilapia and skate) and collagen types (type I and type II) were conducted by SWATH-MS and orthogonal partial least squares discriminant analysis (OPLS-DA). It could provide a theoretical basis for the application of collagen from different types and sources.

## 2. Results

### 2.1. Distribution of Collagen Fibers in TI-s, TI-b, SK-s, and SK-c 

**Figure 1:**
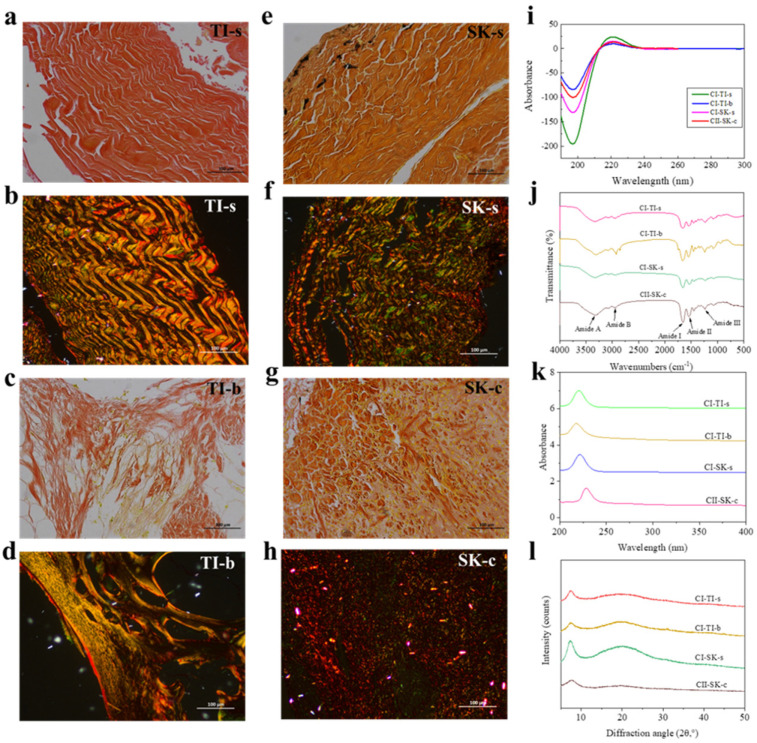
The VG staining and Sirius red staining of collagen in TI−s (**a**,**b**), TI−b (**c**,**d**), SK−s (**e**,**f**), SK−c (**g**,**h**); CD spectra of collagen isolated from tilapia and skate (**i**); FTIR spectra of collagen isolated from tilapia and skate (**j**); UV spectra of collagen isolated from tilapia and skate (**k**); XRD of collagen isolated from tilapia and skate (**l**).

VG staining is usually used to analyze the arrangement and distribution of collagen fibers. VG staining of TI-s (a), TI-b (c), SK-s (e), and SK-c (g) is shown in Figure 1. It can be seen that the red collagen fibers are aligned tightly and longitudinally in TI-s, SK-s, and TI-b. The collagen fibers in the SK-c tissue showed a tight reticular structure.

With Sirius red staining under cross-polarized light, collagen I shows strong red or yellow, collagen II displays multiple colors, and collagen III shows weak green [18]. The arrangement and distribution of collagen in TI-s (b), TI-b (d), SK-s (f), and SK-c (h) under polarized light are shown in Figure 1. The collagen of TI-s, TI-b, and SK-s showed bright yellow and red, and might therefore be type I collagen. In addition, there were also some green collagens interlaced with the bright yellow and red, which may be type III collagen. The collagen of SK-c showed weak birefringence and a variety of colors, and was presumed to be type II collagen. Acid-soluble and pepsin-soluble tilapia skin collagen were characterized as type I collagen [19]. Hwang et al. [20] identified that the major collagen in SK-s was type I using SDS–PAGE pattern and peptide maps analysis.

### 2.2. Structural Characteristics of CI-TI-s, CI-TI-b, CI-SK-s, and CII-SK-c

#### 2.2.1. Analysis of the UV Spectra of CI-TI-s, CI-TI-b, CI-SK-s, and CI-SK-c

The UV absorption spectra of collagen isolated from tilapia skins and bones and skate skins and cartilages are shown in Figure 1k. The maximum absorptions of CI-TI-s, CI-TI-b, CI-SK-s, and CII-SK-c were 222, 218, 221, and 229 nm, respectively. Reátegui-Pinedo et al. [1] found that the UV absorption peak of tilapia skin collagen was at 232 nm. The maximum absorption peak of skate cartilage collagen was at 230 nm in UV [4]. Results showed that the collagen of tilapia and skate had a similar UV absorption characteristic. The presence of tryptophan and tyrosine ensured that the maximum UV absorption wavelength of proteins occurred at 275–280 nm [4]. CI-TI-s, CI-TI-b, CI-SK-s, and CII-SK-c did not display clear absorption peaks at 280 nm, showing that the collagen samples contained a small amount of tryptophan and tyrosine. The UV absorption peak of collagen at about 230 nm was related to the n → Π* transition of C=O, COOH, and CO-NH_2_ groups [4]. The small differences might be related to the amino acid composition. It was found that the tyrosine concentration of skate cartilage collagen was lower than that of tilapia skin collagen [20,21].

#### 2.2.2. FTIR Analysis of CI-TI-s, CI-TI-b, CI-SK-s, and CII-SK-c

The FTIR spectra characteristics of collagen extracted from tilapia and skate are shown in Figure 1j. The amide A band was mainly generated by the N-H stretching vibration [4]; CI-TI-s, CI-TI-b, CI-SK-s, and CII-SK-c were observed at 3336, 3316, 3326, and 3326 cm^−1^, respectively. The amide B band was associated with the asymmetric stretching vibration of CH_2_ [22] and appeared at wavenumbers 2938, 2929, 2935, and 2948 cm^−1^ for CI-TI-s, CI-TI-b, CI-SK-s, and CII-SK-c, respectively. The amide I bands of CI-TI-s (1662 cm^−1^), CI-TI-b (1660 cm^−1^), CI-TI-s (1658 cm^−1^), and CII-TI-c (1652 cm^−1^) was in accordance with the vibration of C=O groups. The amide II of CI-TI-s (1546 cm^−1^), CI-TI-b (1551 cm^−1^), CI-SK-s (1535 cm^−1^), and CII-SK-c (1550 cm^−1^) were similar to the results of Zhu et al. [4]. The amide III bands were associated with the N-H and C-N stretching vibration [4], which of CI-TI-s, CI-TI-b, CI-SK-s, and CII-SK-c were observed at 1236, 1238, 1234, and 1238 cm^−1^, respectively.

#### 2.2.3. CD Spectra Analysis of CI-TI-s, CI-TI-b, CI-SK-s, and CII-SK-c

The CD spectra of CI-TI-s, CI-TI-b, CI-SK-s, and CII-SK-c are shown in Figure 1i. Natural collagen showed a positive peak at 221 nm, a negative absorption at 198 nm, and the intensity ratio of positive and negative absorption peaks (Rpn) of 0.15 [23]. The CD spectra of CI-TI-s, CI-TI-b, CI-SK-s, and CII-SK-c exhibited positive absorption peaks at 221.5, 221.5, 221, and 222 nm, respectively, and negative absorption peaks at 197 nm. The Rpn values were 0.12, 0.12, 0.12, and 0.14, respectively; these values are consistent with the results of Zhu et al. [4]. The CD spectra of collagen from tilapia skins showed a significantly weak positive peak (220 nm) and a strong negative peak (196 nm); the Rpn value of collagen was 0.131 [5].

#### 2.2.4. X-ray Diffraction of CI-TI-s, CI-TI-b, CI-SK-s, and CII-SK-c

The X-ray diffraction (XRD) patterns of CI-TI-s, CI-TI-b, CI-SK-s, and CII-SK-c are shown in Figure 1l. They reveal the two characteristic peaks in the spectra. the first peak, in the range of 5–10°, was associated with the crystal structure inside the collagen [24]. The second peak was about 20° at the diffraction angle (2θ), which was associated with diffuse emission from the internal amorphous region of the collagen [25]. The d values, corresponding to the first peak of CI-TI-s, CI-TI-b, CI-SK-s, and CII-SK-c, were 11.84, 12.20, 12.03, and 11.56 Å, respectively, representing spacing between molecular chains. Sun et al. [23] found d values for the first relatively sharp peak of acid; the pepsin-soluble collagen of tilapia skins were 11.66 Å and 11.90 Å, respectively. The d value, corresponding to the first peak of double-spotted pufferfish skin collagen, was 11.45 Å [26].

### 2.3. The O-Glycopeptide Bond of CI-TI-s, CI-TI-b, CI-SK-s, and CII-SK-c

**Figure 2:**
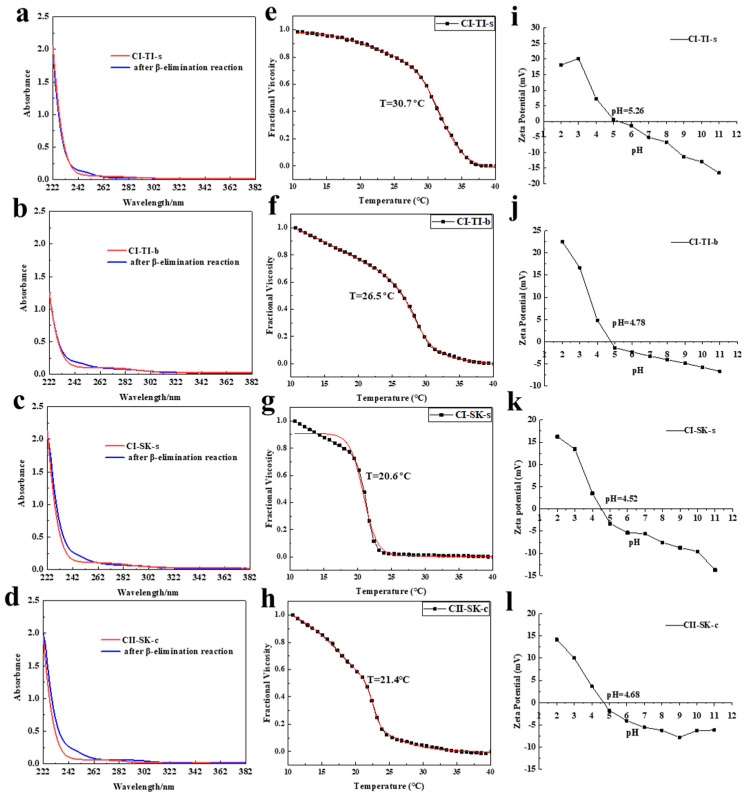
Ultraviolet spectra before and after β elimination reaction, thermal denaturation curves fitting and Zeta potential of CI−TI−s (**a**,**e**,**i**), CI−TI−b (**b**,**f**,**j**), CI−SK−s (**c**,**g**,**k**), and CII−SK−c (**d**,**h**,**l**); The red line were the fractional viscosity fitting curve of collagen.

O-glycosylation, related to the stability of collagen, is formed by the attachment of galactose in its β-configuration to the hydroxyl group of hydroxylysine, which was converted from hydroxylated lysine [4]. The β-elimination reaction (alkali-treatment) can break the O-glycopeptide bond of collagen, which produces more unsaturated amino acids to enhance the UV absorption at 240 nm [27]. The UV spectra before and after β-elimination reaction of CI-TI-s (Figure 2a), CI-TI-b (Figure 2b), CI-SK-s (Figure 2c), and CII-SK-c (Figure 2d) were illustrated in Figure 2. The enhancement of UV absorption after β-elimination reaction of collagen at 240 nm indicated that the O-linked glycopeptide bonds were present, which is consistent with the results of Zhu et al. [4]. O-linked glycopeptide bonds were present in a higher level on CII-SK-c when compared with CI-TI-s, CI-TI-b, and CI-SK-s.

### 2.4. Td and Zeta Potential of CI-TI-s, CI-TI-b, CI-SK-s, and CII-SK-c

The fractional viscosity fitting curve of CI-TI-s (Figure 2e), CI-TI-b (Figure 2f), CI-SK-s (Figure 2g), and CII-SK-c (Figure 2h) are shown in Figure 2. The thermal denaturation temperatures (Td) of CI-TI-s, CI-TI-b, CI-SK-s, and CII-SK-c were 30.7, 26.5, 20.6, and 21.4 °C, respectively. The Td of tilapia skins and bone collagen was higher than that of skate skins and cartilage collagen, and CII-SK-c showed a higher Td when compared with that of CI-SK-s. The thermal denaturation temperatures of different collagen can be affected by the temperature in which the species live. Wang et al. [28] found that the Td in the deep sea was lower than that in the shallow sea. It was reported that the Td temperature of collagen was raised as the content of Hyp increased [29]. More proline and hydroxyproline increased the thermal stability of collagen because of a higher density of crosslinks [30], which can also be improved by chitin nanofibers for the usage as scaffolds and wound-dressing materials [31]. Klabukov et al. [32] found that the copolymer had a higher stability and the ability to resist hydrolysis.

Zeta potential can be used to describe the surface charge of colloidal systems, and the isoelectric point (pI) refers to the pH at which the net charge of molecule is zero [33]. The rate of collagen fibril production is largely controlled by electrostatic interactions, and collagen is more conducive to the aggregation of collagen molecules and fibrils formation when the collagen pI is reached [34]. The zeta values of four collagen proteins are shown in Figure 2. The pI of CI-TI-s (Figure 2i), CI-TI-b (Figure 2j), CI-SK-s (Figure 2k), and CII-SK-c (Figure 2l) were 5.26, 4.78, 4.52, and 4.68, respectively, which may be due to the high content of acidic amino acids (glutamate and aspartic acid) in collagen. Ahmed et al. [33] found the pI of collagen might be associated with amino acid composition and amino acid residue sequences, especially on surface domains.

### 2.5. The Self-Assembly Properties of CI-TI-s, CI-TI-b, CI-SK-s, and CII-SK-c

**Figure 3:**
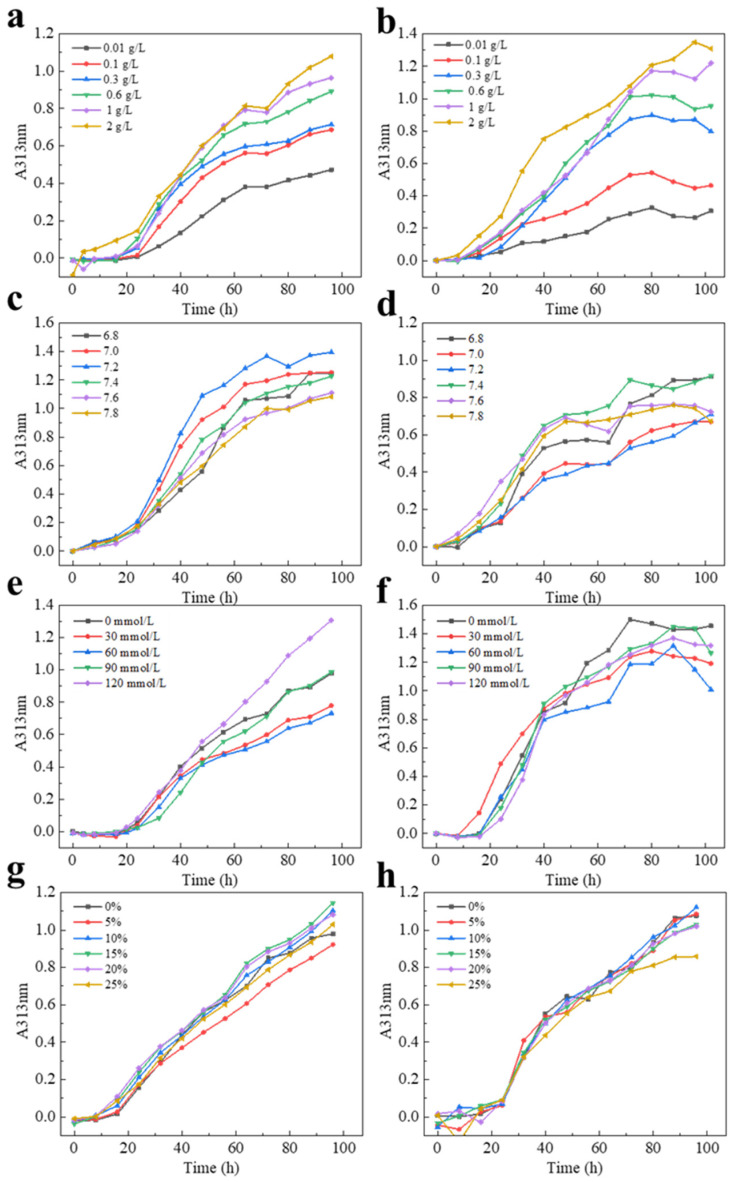
Effects of concentration (**a**,**b**), pH (**c**,**d**), ion strength (**e**,**f**), and HA (**g**,**h**) induce on the kinetic of collagen self-assembly of CI-TI-s and CII-SK-c.

The effects of concentration, pH, ionic strength, and hyaluronic acid (HA) on the kinetics of collagen self-assembly of CI-TI-s and CII-SK-c are shown in Figure 3. The degree of collagen self-assembly was positively correlated with the concentration of collagen (Figure 3a,b). The higher self-assembly of the CI-TI-s (Figure 3c) was at pH 6.8, and the self-assembly of CII-SK-c (Figure 3d) in the range of pH 7.4–7.6 was higher than that at other pH values, which was similar to the results of Yan et al. [35]. Figure 3e showed that the aggregation rate of CI-TI-s was increased when the sodium chloride concentration reached 80 mmol/L. The aggregation rate of CII-SK-c showed similar characteristics at different ionic strengths (Figure 3f). The self-assembly of CI-TI-s could be found when the HA concentration was in the range of 10% to 20%. However, the self-assembly behavior of CII-SK-c could not be affected by HA (Figure 3g,h). At pH 7.4–7.6 and NaCl concentration of 120 mmol/L, collagen in skate and sturgeon cartilage at concentrations above 0.3 mg/mL exhibited significant self-assembly behavior [4]. In summary, the self-assembly characteristics of CI-TI-s and CII-SK-c were different.

### 2.6. Discovery of Unique Peptides for C-TI and C-SK

**Figure 4:**
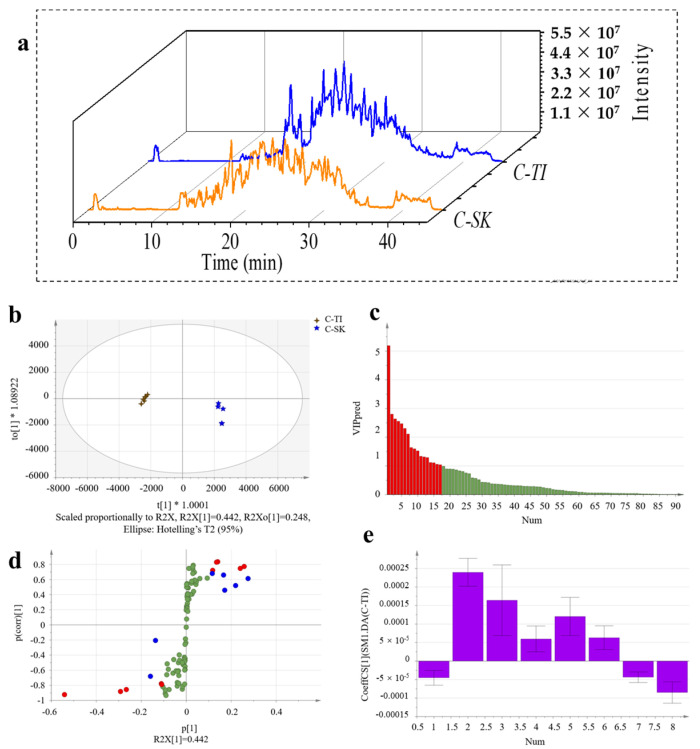
SWATH−MS data analysis of collagen peptides. Representative total ion chromatograms (TICs) with SWATH data of C−TI (blue) and C−SK (orange) (**a**). The OPLS−DA (**b**) (the blue were C−SK and the brown were C−TI; “*” was weights of regression coefficient), VIP (**c**) (the red rectangles were peptides with a VIP value > 1), S−plot (**d**) (the green were VIP value < 1; the blue were VIP value > 1 but |p(corr)| < 0.7; the red were VIP value > 1 and |p(corr)| > 0.7), and Knife cut confidence interval analysis (**e**) for discovering peptide markers of C−TI versus C−SK.

The mass spectrum information of C-TI and C-SK was obtained using DDA collection mode. The DDA data from two samples were searched, and 698 peptides and 17 proteins (C-TI) and 331 peptides and 31 proteins (C-SK) were found at the 95% confidence level. The score plot of OPLS-DA in Figure 4b demonstrated that collagen samples from tilapia were thoroughly separated with those from skate, indicating potential of SWATH-MS based proteomic analysis in discrimination of collagen sources. The OPLS-DA evaluation model parameters consist of R^2^X, R^2^Y, and Q^2^, where R^2^X, and R^2^Y stand for the explanation rate of the model for the X and Y matrices, respectively, while Q^2^ indicates the predictive power of the model. For a perfect categorization, the value of specificity should be near to 1 [36]. R^2^X, R^2^Y, and Q^2^ of the model were 0.712, 0.998, and 0.985, respectively. 

The peptides with a variable importance in the projection (VIP) value higher than 1 were screened. Figure 4c showed that there were 17 peptides with a VIP value > 1 (marked in red) that could be used as potential identification peptides for subsequent analysis. Then the peptides with |p(corr)| > 0.7 in S-plot were selected to the next screen (Figure 4d). The farther from the origin in the S-plot score chart, the greater the contribution of this point to sample classification. Finally, eight specific peptides (marked with purple in Figure 4e) with the jack-knifed confidence intervals in the coefficients plot not passing through zero were identified as potential marker peptides of C-TI and C-SK. Statistical analysis showed that there were significant differences between two samples. To perform the biospecificity check, the bioinformatics tool, Blast, was used to perform a peptide amino acid sequence search of the entire NCBI protein sequence database [37]. A total of eight candidate tagged peptides were submitted to Blast search for biospecificity checks against the entire NCBI non-redundant protein database. The unique peptides of C-TI and C-SK were revealed through the proteomic and chemometrics analysis, including GPSGPQGAVGATGPK, PAMPVPGPMGPMGPR, SPAMPVPGPMGPMGPR, GESGPSGPAGPAGPAGVR, SSGPPVPGPIGPMGPR, GLTGPIGVPGPPGAQGEK, GLAGPQGPR, and GLSGDPGVQGIK (Table 1). The detectability assessment of the potential marker peptides can be performed using the MRM approach. The 81 proteins of skipjack tuna, bigeye tuna, and yellowfin tuna were quantified by SWATH-MS proteomic analysis, and 14 potential protein biomarkers were screened by chemometrics [17]. Results showed that it was feasible to screen unique peptides for C-TI and C-SK using the SWATH-MS proteomic approach.

### 2.7. Discovery of Unique Peptides for Type I and Type II Collagen

**Figure 5:**
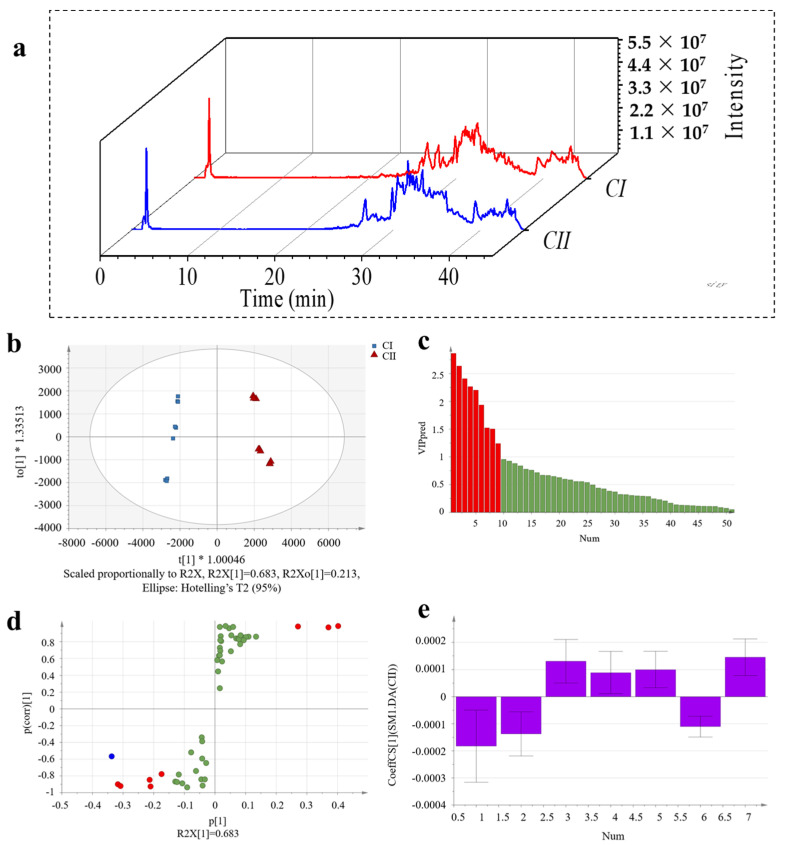
SWATH−MS data analysis of collagen peptides. Representative total ion chromatograms (TICs) with SWATH data of CI (red) and CII (blue) (**a**). The OPLS-DA (**b**) (the blue were CI and the red were CII; “*” was weights of regression coefficient), VIP (**c**) (the red rectangles were peptides with a VIP value > 1), S−plot (**d**) (the green were VIP value < 1; the blue were VIP value > 1 but |p(corr)| < 0.7; the red were VIP value > 1 and |p(corr)| > 0.7), and Knife cut confidence interval analysis (**e**) for discovering peptide markers of CI versus CII.

The analysis of unique peptides for type I and type II collagen are shown in Figure 5. The mass spectrum information of CI and CII was obtained using the DDA collection mode, and the DDA data from two samples were searched, resulting in 275 peptides and 24 proteins (CI) and 144 peptides and 16 proteins (CII) at the 95% confidence level; these were taken as the quantitative database of SWATH-MS for subsequent analysis. The OPLS-DA analysis was performed to seek marker peptides as differentiators indicating types from collagen I and collagen II. R^2^X, R^2^Y, and Q^2^ of the OPLS-DA evaluation model were 0.895, 0.983, and 0.985, respectively. Figure 5c showed that there were nine peptides with a VIP value >1 (marked in red). There were seven specific peptides (marked with purple in Figure 5e) with the jack-knifed confidence intervals in the coefficients plot not passing through zero. In addition, unique peptides for CI and CII were identified, including GPTGEIGATGLAGAR, GVLGLTGMR, LGLTGMR, GEPGAAGPAGPSGPMGPR, SSGPPVPGPIGPMGPR, and GLSGDPGVQGIK (Table 1). The results showed the ability to search for unique peptides for type I and type II collagen using the SWATH-MS proteomic approach.

## 3. Materials and Methods

### 3.1. Materials

Skins and bones of tilapia (*Oreochromis mossambicus*) and skins and cartilages of skate (*Raja porosa*) were obtained from Nanshan Aquatic Products (Qingdao, China). All raw materials were preserved at −20 °C. Acetonitrile was obtained from Merck (Darmstadt, Germany). Trypsin was obtained from Promega Co., Ltd. (Beijing, China). The pepsin was obtained from Maclean Biochemical Technology Co., Ltd. (Shanghai, China). The other reagents were of analytical grade and bought from Sinopharm Chemical Reagent Co., Ltd. (Shanghai, China).

### 3.2. Van Gieson and Picric Acid-Sirius Red Staining

Tilapia skin (TI-s), tilapia bone (TI-b), skate skin (SK-s), and skate cartilage (SK-c) were treated in 4% formaldehyde for two days and then washed with water. Samples were dehydrated, embedded in paraffin, and then stained with Van Gieson (VG) and Picric acid-Sirius red. Tissues stained with Picric acid-Sirius red were observed using a cross-polarized light microscope and tissues stained with VG were observed using an ordinary light microscope (Ni-E, Nikon Imaging Instrument Sales Co., Ltd., Shanghai, China). For tilapia bones, their pretreatment should be decalcified in a 10% EDTA-Na2 solution for two weeks before dehydration.

### 3.3. Preparation of Collagen

Samples were washed with deionized water (4 °C) after being removed the residual meat with a knife and divided into small pieces (1 cm × 1 cm). They were then soaked for 48 h (1:20, *w*/*v*) with the 0.1 mol/L concentration of NaOH. After discarding the non-collagen components, fish skins were retreated with the pre-cold deionized water for a neutral pH. Then the pretreated materials were poured into acetic acid solution (0.5 mol/L) that contained pepsin with the concentration of 0.1% (*w*/*v*). After being stirred for 48 h at 4 °C, it was centrifuged (9000× *g*) for 20 min at 4 °C (2–16KL, Sigma, An Der Unteren Söse 50, Osterode Am Harz, Germany). The collagen was sedimented by adding NaCl to the supernatants until its concentration reached 0.9 mol/L. The collagen was gathered by centrifugation at 6000× *g* for 30 min and redissolved in 0.5 mol/L acetic acid solution. Then the mixture was dialyzed and lyophilized (VSP62, Marin Christ Co., An Der Unteren Söse 50, Osterode Am Harz, Germany) to obtain tilapia skin collagen (CI-TI-s), skate skin collagen (CI-SK-s), and skate cartilage collagen (CII-SK-c). For tilapia bone collagen (CI-TI-b), the extraction method was similar. Before using acetic acid and pepsin to extract collagen from tilapia bones, the bones needed to be pretreated with EDTA-Na_2_ (0.5 mol/L) for 48 h to remove the calcium.

### 3.4. Fourier Transform Infrared (FTIR) Spectroscopy Analysis

FTIR was detected according to the method of Zhu et al. [4]. Freeze-dried collagen was thoroughly mixed, ground with dehydrated potassium bromide, and pressed into sheets. FTIR was measured at 4000–400 cm^−1^ (Nicolet iS 10, Thermo Fisher Scientific, Waltham, MA, USA). It was scanned at the resolution of 2 cm^−1^ 64 times and OMNIC 9.0 software was used for the analysis.

### 3.5. Determination of Ultraviolet-Visible Absorption Spectra

The 0.5 mg/mL samples of collagen were prepared with 0.1 mol/L acetic acid and centrifuged at 9000× *g* for 5 min at 4 °C. They were scanned by an ultraviolet spectrophotometer (UV-2102 PC, Unico Co., Ltd., Shanghai, China) in the range of 190–400 nm [4].

### 3.6. Circular Dichroism (CD) Spectra Analysis

The 0.5 mg/mL samples of collagen were prepared with 0.05 mol/L acetic acid. After filtering through a 0.22 μm membrane, the solution was measured in the scanning range of 190–260 nm [23].

### 3.7. Determination of X-ray Diffraction (XRD)

The XRD patterns were determined with Cu Kα radiation applied at 40 kV of voltage. The samples were recorded with a scanning angle (2θ) from 5 ° to 40 ° at a scanning rate of 4 °min^−1^. The d value can be calculated by using the Bragg’s equation as follows [23]:

$$d\;(\text{Å})=\frac{\lambda }{2\sin\theta }$$

where θ and λ were the Bragg diffraction angle and X-ray wavelength, respectively.

### 3.8. Thermal Denaturation Temperature (Td) Analysis

The 4‰ collagen was prepared using a 0.1 mol/L acetic acid solution. The viscosity of the solution was analyzed from 4 °C to 40 °C using a rheometer (MCR301, Anton Paar GmbH Co., Ltd., Graz, Austria) at the heating rate of 0.4 °C/min. The Td was computed, at which the fractional viscosity was 0.5 [38]. The specific calculation formula was as follows:

$$F(T)=\frac{\eta_{sp}\left(T\right)-\eta_{sp}\left(40\;{}^{\circ}C\right)}{\eta_{sp}\left(4{}^{\circ}C\right)-\eta_{sp}\left(40\;{}^{\circ}C\right)}$$

where F was fractional viscosity, η_sp_ was computed by $\frac{\eta -\eta_{0}}{\eta_{0}}$, η was the viscosity of the solution, and η_0_ was the viscosity of acetic acid (mPa·s).

### 3.9. Zeta Potential Measurement

Zeta potential was measured according to the method of Yan et al. [35]. The pH of 0.5 mg/mL collagen was adjusted to 2–11 and it was measured using a nano size potentiometer (ZS90, Malvern Instruments, Malvern, UK).

### 3.10. LC/MS Analysis of Collagen of Different Types and Sources

For the discovery of unique collagen peptides of different sources, collagen from tilapia skins (CI-TI-s), tilapia bones (CI-TI-b), skate skins (CI-SK-s), and skate cartilages (CII-SK-c) were digested with sequencing grade trypsin at 37 °C for 18 h at a protein/trypsin ratio of 25:1 (*w*/*w*). After centrifugation, the digested samples of CI-TI-s and CI-TI-b were mixed in equal volumes, named as C-TI. The digested samples of CI-SK-s and CII-SK-c were mixed in equal volumes, named as C-SK. 

For the discovery of unique collagen peptides of different types, the treatment of collagen from cod skins (CI-GA-s), cod bones (CI-GA-b), sturgeon skins (CI-ST-s), and sturgeon cartilages (CII-ST-c) was the same as above. The digested samples of CI-GA-s, CI-TI-s, CI-ST-s, CI-SK-s, CI-GA-b, and CI-TI-b were mixed in equal volumes, named as CI. The digested samples of CII-ST-c and CII-SK-c were mixed in equal volumes, named as CII. 

Sample separation was performed by mass spectrometry using an UHPLC-Q/TOF instrument in DDA mode equipped with AdvanceBio Peptide Map column (150 mm × 2.1 mm, 130 Å, 2.7 µm), and it was operated in electrospray positive ion (ESI+) mode with a spray voltage of 5500 V in range of 350–1500 m/z. The data were identified by Protein Pilot software (version 5.0.2, SCIEX) to generate the library for the SWATH analysis. Data from SWATH acquisitions were imported by PeakView software (version 2.1, SCIEX), and then normalized based on the total peak areas sum by MarkerView software (version 1.2.1, SCIEX). Orthogonal partial least squares discriminant analysis (OPLS-DA) was used to screen peptides between CI and CII or C-SK and C-TI in SMICA software (version 14.0, Umetrics). The specificity verification of potential peptide markers was achieved by the Blast searching of the whole NCBI protein database [39].

Concretely, the mass spectrum information was obtained using DDA collection mode, and the Protein Pilot software was used to search the database. In the process of mass spectrometry analysis, the DDA data were searched at the 95% confidence level, which were taken as the quantitative database of SWATH-MS for subsequent analysis. The data obtained by SWATH-MS are quite complex. A further decoding process is necessary for generating meaningful information. The different peptides with a false positive error rate of less than 1% were selected for subsequent OPLS-DA analysis using SIMCA software [40]. To seek marker peptides as differentiators indicating collagen sources, the separated groups were classified as one group and OPLS-DA analysis was then performed.

### 3.11. Statistical Analysis

Each experiment was repeated at least three times. Statistical analyses were performed using one-way analysis of variance (ANOVA). The probability value of *p* < 0.05 was used as the criterion for significant differences.

## 4. Conclusions

In this study, the distribution of collagen fibers in tissues of tilapia skin and bone and skate skin fish and cartilage was different. All collagen had a complete triple helix structure and similar UV and X-ray characteristics. Type II collagen had obvious glycopeptide linkage. The Td temperature, Zeta potential, and the optimal self-assembly conditions of collagen from different sources were different. Further studies have shown that unique peptides of different sources and types of collagens could be obtained by SWATH-MS.

## Figures and Tables

**Table 1:** All unique peptides screened by chemometrics.

	Peptide Sequence	*m/z*	Protein Source
C-TI/C-SK	data		data
1	GPSGPQGAVGATGPK	640.8333	Type I procollagen alpha 1 chain OS = Okamejei kenojei GN = SkCOL1A1 PE = 2 SV = 1
2	PAMPVPGPMGPMGPR	746.3671	Uncharacterized protein (Fragment) OS = Oreochromis niloticus GN = col1a1 PE = 4 SV = 1
3	SPAMPVPGPMGPMGPR	789.8831	Uncharacterized protein (Fragment) OS = Oreochromis niloticus GN = col1a1 PE = 4 SV = 1
4	GESGPSGPAGPAGPAGVR	760.8762	Uncharacterized protein OS = Oreochromis niloticus GN = LOC100694532 PE = 4 SV = 1
5	SSGPPVPGPIGPMGPR	751.8928	Uncharacterized protein OS = Oreochromis niloticus GN = LOC100694532 PE = 4 SV = 1
6	GLTGPIGVPGPPGAQGEK	816.4412	Uncharacterized protein OS = Oreochromis niloticus GN = LOC100694532 PE = 4 SV = 1
7	GLAGPQGPR	426.7379	Uncharacterized protein (Fragment) OS = Anolis carolinensis GN = COL1A2 PE = 4 SV = 1
8	GLSGDPGVQGIK	564.3064	Uncharacterized protein OS = Gasterosteus aculeatus PE = 4 SV = 1
CI/CII			
1	GPTGEIGATGLAGAR	664.3519	Collagen type I alpha 2 OS = Oreochromis niloticus GN = COL1A2 PE = 2 SV = 1
2	GVLGLTGMR	452.2577	Type I procollagen alpha 1 chain OS = Okamejei kenojei GN = SkCOL1A1 PE = 2 SV = 1
3	LGLTGMR	374.2127	Type I procollagen alpha 1 chain OS = Okamejei kenojei GN = SkCOL1A1 PE = 2 SV = 1
4	GEPGAAGPAGPSGPMGPR	781.8726	Type I procollagen alpha 1 chain OS = Okamejei kenojei GN = SkCOL1A1 PE = 2 SV = 1
5	SSGPPVPGPIGPMGPR	751.8928	Uncharacterized protein OS = Oreochromis niloticus GN = LOC100694532 PE = 4 SV = 1
6	GLSGDPGVQGIK	564.3064	Uncharacterized protein OS = Gasterosteus aculeatus PE = 4 SV = 1

## Data Availability

The data presented in this study are available on request from the corresponding author.

## Sample Availability

**Available.**
